# Deficits in Upper Limb Long Lever Isometric Force After Shoulder Stabilization Surgery in Australian Rules Footballers

**DOI:** 10.1177/23259671251342585

**Published:** 2025-07-03

**Authors:** Peter K. Edwards, Nicholas Blackah, Jo McEwan, Peter D’Alessandro, Jay R. Ebert

**Affiliations:** † School of Allied Health, Faculty of Health Sciences, Curtin University, Bentley, Western Australia, Australia; ‡ Orthopaedic Research Foundation of Western Australia, Perth, Western Australia, Australia; § Fiona Stanley Fremantle Hospitals Group, South Metropolitan Health Service, Perth, Western Australia, Australia; || School of Surgey, University of Western Australia, Crawley, Western Australia, Australia; ¶ School of Human Sciences, University of Western Australia, Crawley, Western Australia, Australia

**Keywords:** shoulder stabilization, contact athletes, isometric strength, ASH test

## Abstract

**Background:**

Early surgical interventions are common for traumatic anterior shoulder instability in contact and collision sport athletes. It is unclear which tests, and which criteria, should be used to guide return to sport after surgery. As such, additional knowledge on postoperative shoulder function is needed to guide this decision making.

**Purpose:**

To assess deficits in upper limb long lever isometric force and the rate of force development (RFD) in Australian rules footballers after anterior shoulder stabilization surgery.

**Study Design:**

Cross-sectional study; Level of evidence, 3.

**Methods:**

A total of 30 male Australian rules footballers who had undergone unilateral anterior shoulder stabilization surgery were assessed at 4 to 6 months postoperatively. An uninjured age-, sex-, and sport-matched control group (n = 30) was assessed on a single occasion. Isometric peak force and RFD (within the first 100 and 200 milliseconds) were measured using the Athletic Shoulder (ASH) test in 3 positions (ISO-I, ISO-Y, and ISO-T). Data were analyzed for interlimb differences within the surgical group and between-group differences against controls. The reliability of the measurements was also assessed, and correlations between peak force and RFD were determined.

**Results:**

In all ASH test positions, the surgical arm showed significantly lower peak force and RFD within 200 milliseconds than both the nonsurgical arm and control group (*P* < .05). No differences were found in the RFD within 100 milliseconds (*P* > .05). Large effect sizes were noted in peak force deficits between the surgical arm and control group, particularly in the ISO-T position (*d* = 1.19; *P* < .001). Interlimb asymmetries were highly variable and did not consistently favor the nonsurgical arm.

**Conclusion:**

Australian rules footballers at 4 to 6 months after shoulder stabilization surgery exhibited significant deficits in long lever isometric force and late-phase RFD. Clinicians should be cautious in interpreting limb asymmetry and RFD measurements during return-to-sport assessments. Further research should explore the long-term outcomes and relationship between these physical measurements, patient-reported outcomes, and reinjury rates.

Anterior shoulder instability is common and often a result of traumatic injuries to the upper limb in overhead and contact sports.^8^ Australian rules football is a fast-paced contact sport involving multidirectional tackling and overhead movements that impose high stress on the shoulder. The most common mechanism of shoulder dislocations in this sport occurs at <100° of flexion/abduction, such as tackling another player.^8,28^ Contact and collision sports, such as Australian rules football, tend to have a higher rate of recurrent shoulder instability than other sports.^2^ Early surgical interventions are often preferred over nonoperative management to increase the likelihood of timely return to sport (RTS) and minimize the chances of recurrent instability.^9,12,13,18^ Current evidence suggests that athletes achieve RTS at approximately 6 months after anterior shoulder stabilization surgery, irrespective of the type of surgical procedure.^10,16^ However, this may be limited to training (modified or unrestricted) activities or competing at levels below their preinjury level in which they may not be deemed “ready” to return to their preinjury level of competition or performance.^3^ Furthermore, despite being cleared to RTS, contact and collision sport athletes continue to demonstrate upper limb strength and performance deficits compared with their nonoperative arm or their uninjured counterparts.^14^

To assist in the decision on an athlete’s readiness to RTS, a battery of tests, which may include patient-reported outcome measures, clinical examinations, and sport-specific functional assessments, is recommended.^16^ However, for the athlete returning to sport after shoulder stabilization surgery, there is currently no gold-standard test, or battery of tests, to aid in these decisions.^29^ Shoulder range of motion (ROM) and rotation strength are the most frequently assessed after surgery.^16^ Isokinetic dynamometry, while having shown good reliability in uninjured collision and contact athletes,^15^ is often limited in its accessibility, while isometric strength testing using a handheld dynamometer is prone to measurement errors.^5^ However, in sports such as Australian rules football that require multidirectional tackling and overhead tasks, which are common mechanisms of injury,^8,28^ assessing an athlete’s ability to withstand long lever stress and to produce force, and produce it quickly, may be critical. Assessments of isometric peak force with the shoulder in long lever positions have been suggested to be used in an RTS battery of tests for contact and collision athletes after injuries or surgery^4,29^ and have previously demonstrated good reliability.^5^ However, to our knowledge, their use in Australian rules footballers, both healthy players and those after anterior shoulder stabilization, has yet to be reported.

The aim of this study was to assess deficits in upper limb long lever isometric force and the rate of force development (RFD) using the Athletic Shoulder (ASH) test in patients after anterior shoulder stabilization surgery for injuries sustained during Australian rules football. The study also sought to explore the relationship between RFD and peak force and to examine limb asymmetry in these measurements. We hypothesized that patients would exhibit significant deficits in peak force and RFD in the operated arm compared with the uninvolved arm and an uninjured, matched control group. Additionally, we hypothesized that the RFD at 0 to 100 milliseconds and at 0 to 200 milliseconds would be strongly correlated with peak force across all long lever isometric force test positions.

## Methods

### Study Design and Participants

This cross-sectional study recruited a consecutive series of patients (n = 30), referred by a single orthopaedic surgeon with fellowship training in sports surgery (P.D.), for a single functional assessment in a private clinic between 4 and 6 months after shoulder stabilization surgery. Patients who underwent shoulder stabilization surgery between May 2021 and March 2024 were considered for study eligibility. Patients were included if they were male, aged between 16 and 45 years, participated in Australian rules football at a subelite level (defined as tier 2 or 3 athletes),^21^ and underwent Bankart repair (with or without remplissage) or the open Latarjet procedure for the management of a traumatic injury sustained during competitive Australian rules football. Patients were excluded if they had multidirectional shoulder instability, other shoulder abnormalities associated with shoulder instability (such as a rotator cuff tear), a recurrence of dislocations or subluxations since surgery, or the presence of poor bony union assessed via computed tomography at 3 months for those who underwent the Latarjet procedure. Patients with pre-existing conditions related to upper extremity pain (such as cervical neuropathy or other neurological pain) were also excluded. Patients were also excluded if they had postoperative shoulder stiffness and were not able to achieve full shoulder flexion and horizontal abduction. A convenience sample of uninjured male Australian rules footballers (n = 30) was recruited from local subelite clubs between June and August 2024. This control group was matched by age, sex, sport (Australian rules football), and competition level (subelite). Players were considered eligible to be included in this control group if they reported no history of shoulder instability or upper limb surgery and met the same inclusion criteria as the surgical group. All participants gave informed written consent before testing, and the study received ethical approval from Curtin University’s Human Research Ethics Committee and the University of Western Australia’s Human Research Ethics Committee.

### Surgical Techniques

Patients in this study underwent anterior stabilization through 1 of 3 procedures: arthroscopic Bankart repair, arthroscopic Bankart repair with remplissage, or the open Latarjet procedure. Surgical technique selection was individualized based on factors such as sport type, patient and club preferences, and glenoid bone loss. Patients with glenoid off-track lesions or >10% bone loss were treated with the Latarjet procedure, while a small subset of patients without measurable bone loss also underwent the Latarjet procedure based on sport-specific demands and preferences. Patients who underwent arthroscopic Bankart repair were initially examined under anesthesia and positioned laterally. A 3-portal arthroscopic technique was then employed, and once the Bankart lesion was appropriately elevated and reduced, arthroscopic repair was initiated. A luggage-tag horizontal mattress suture configuration was employed, ensuring at least 6 suture passes into 3 knotless anchors placed along the anteroinferior glenoid rim to achieve effective capsular shift accompanying labral repair. Arthroscopic Bankart repair with remplissage was selected for patients considered to have a higher risk of instability or in the presence of a Hill-Sachs lesion that was engaging and required manual reduction during the examination under anesthesia. There were 2 knotted anchors placed 5 mm from the articular rim of the Hill-Sachs lesion, followed by posterior capsulodesis after Bankart repair. The Latarjet procedure was performed by initially positioning the patient in a modified beach-chair position. A diagnostic arthroscopic examination was conducted to evaluate the articular cartilage’s condition, address any concurrent abnormalities (eg, a SLAP tear), and confirm the extent of glenoid or humeral bone loss. Following a deltopectoral approach, osteotomy was conducted at the base of the coracoid. The conjoint tendon was then mobilized, and the subscapularis and capsule were split for glenoid access. After preparing the bed of the anteroinferior glenoid, the coracoid was affixed to the glenoid using 2 solid 4-mm titanium screws, and the shoulder was supported, ensuring that the dynamic sling effect of the conjoint tendon conferred appropriate stability while maintaining ROM.

### Postoperative Follow-up and Rehabilitation

Postoperative clinical follow-up was undertaken at approximately 2 weeks, 8 weeks, and 4 months after surgery by the operating surgeon (P.D.), at which any recurrence of anterior subluxations or dislocations was assessed, alongside any complications, shoulder pain beyond what may be expected, shoulder mobility, clinical signs of apprehension, and strength. For those patients who underwent the Latarjet procedure, computed tomography was performed at 3 to 4 months to confirm bony union. Once these criteria had been satisfied, patients were referred for the functional assessment, which was performed between 4 and 6 months after surgery.

Patients were provided with generic rehabilitation advice and a standard physical therapy protocol from their surgeon, which were undertaken in consultation with their physical therapist. After all procedures, the patients were placed in a standard broad arm sling for 4 weeks. Early pendulum and passive ROM exercises were encouraged, along with external rotation limited to 10°. Active ROM was then commenced around 4 weeks after surgery, with strengthening exercises focused on the glenohumeral and scapular stabilizers, beginning once approximately 80% of the patient’s ROM had been achieved. While the timing of RTS was a shared decision made between the player, surgeon, and physical therapist, all patients were provided guidance that RTS could be considered at a minimum of 4 months postoperatively and when 90% limb symmetry in ROM and perceived restoration of preoperative strength were achieved.

### ASH Test

The ASH test was undertaken to assess isometric peak force and RFD of the operated and nonoperated limbs in the surgical group as well as the dominant and nondominant limbs in the uninjured control group. These measurements were collected using portable, uniaxial dual force plates (ForceDecks FDLite [Version 2]; VALD), which recorded vertical ground-reaction force at 1000 Hz. The force plates were connected to a desktop computer running ForceDecks software for Windows (VALD). ForceDecks has shown high accuracy compared with laboratory-grade force plates for various performance tests and variables, including moderate to excellent validity for the ISO-I, ISO-Y, and ISO-T positions when assessing peak vertical force and RFD at 200 milliseconds.^11^

**Figure 1.**
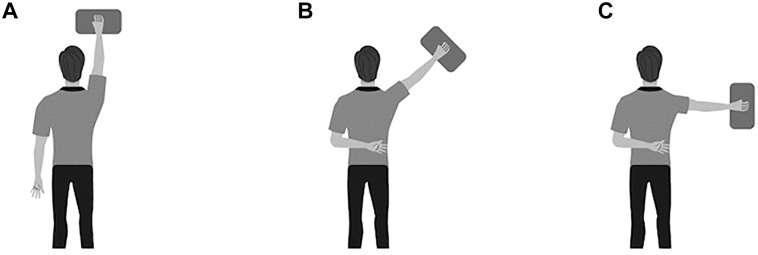
Long lever isometric maximum voluntary contraction (MVC) positions on the Athletic Shoulder (ASH) test, with varying shoulder abduction angles: (A) ISO-I: shoulder in 180° of abduction, (B) ISO-Y: shoulder in 135° of abduction, and (C) ISO-T: shoulder in 90° of abduction.

The ASH test was performed per protocols that have been previously described.^5^ Participants were asked to lie prone on the floor with their hand placed on one of the dual force plates. Patients were asked to complete the following in order: (1) ISO-I position in which the shoulder was in full abduction (in line with the body) and the elbow was fully extended, with the contralateral arm placed down by the side; (2) ISO-Y position in which the arm was in 135° of shoulder abduction and the elbow was fully extended, with the contralateral arm placed at the small of the back; and (3) ISO-T position in which the arm was in 90° of shoulder abduction and the elbow was fully extended, with the contralateral arm placed at the small of the back (Figure 1). In all positions, the forearm was pronated (ie, the palm flat on the force platform), and the heel of the hand acted as the main contact point at the center of the force platform without any finger elevation.

Before the ASH test at maximal effort, all participants were subjected to a standardized warm-up consisting of self-selected stretches, light resistance band motion using the shoulder, and after a demonstration of the protocol, 2 submaximal efforts in each of the ASH test positions, which also acted to familiarize them with the protocol. After zeroing the force plates and weighing the patient’s upper limb, 3 trials were conducted for each limb in the ISO-I position. Each test position always began with the right arm, followed by the left, regardless of whether the arm was the dominant or nondominant limb (dominant arm defined as the one used for throwing) or whether the arm was the operated or nonoperated limb. This sequence was repeated for the ISO-Y and ISO-T positions. All patients were given standardized instructions and consistent verbal encouragement during each trial, with a countdown before each trial. Specifically, patients were asked to “push through the middle of the plate with the palm of your hand and keep your hand flat, as fast and as hard as possible” for a full 3-second duration to capture maximal force (peak force) as fast as possible (RFD). A 20-second rest period was provided between each trial.

### Data Processing

Data from both force plates were analyzed with ForceDecks software using default settings. ForceDecks software automatically detected the initiation of the movement once the test had commenced. Specifically, the start of the movement during the ASH test was determined by applying a low-pass filter of 5 Hz, with the initial location identified at the beginning of the maximal RFD over a 200-millisecond window and refined by a yank threshold of 40 N/s. Each trial was visually inspected for test quality, with the exclusion of any misidentified or incorrectly performed trials (eg, if the hand lifted from its resting position on the platform before pushing down, employing a compensatory strategy to achieve greater force, or performing the test with obvious low effort). The mean value of the 3 trials for each test position for each limb was recorded and used for subsequent analysis. Overall, 4 vertical ground-reaction force outputs from the ASH test, which have been previously reported,^4,5,11^ were selected in this study. Variables of interest included peak force (peak force achieved during the trial minus baseline force on the platform before the start of the movement) and RFD at 0 to 100 milliseconds and 0 to 200 milliseconds. Interlimb asymmetry was calculated by standard percentage differences, along with the use of the “IF function” in Excel (Microsoft), which considers both the magnitude and direction of asymmetry.^6^ The asymmetry index (AI) was calculated using the following equation:

$$\begin{array}{l} \mathrm{Asymmetry}\;\mathrm{Index=}\\ \left(\frac{\mathrm{non-surgical}\;\mathrm{arm}\;\mathrm{minus}\;\mathrm{surgical}\;\mathrm{arm}}{\mathrm{non-surgical}\;\mathrm{arm}}\right)\times 100\\ \times \mathrm{IF}\;(\mathrm{non}-\mathrm{surgical}\;\mathrm{arm}<\mathrm{surgical}\;\mathrm{arm},1,-1). \end{array}$$

Using the aforementioned equation, a positive AI indicates that the value is greater for the uninvolved arm, and a negative AI indicates that the value is greater for the involved (surgically stabilized) arm.

### Statistical Analysis

The normality of the distribution of data was assessed with the Shapiro-Wilk test. Descriptive statistics (mean ± standard deviation) for all variables were calculated. The absolute between-trial reliability of each variable in each position/limb was evaluated using 2-way mixed-effects intraclass correlation coefficients with 95% confidence intervals as well as the coefficient of variation (CV). The CV was calculated for each participant for each position (and limb) and then averaged across all participants. The between-trial reliability was considered acceptable if the CV was ≤10%^30^ and was further categorized as “excellent” if the reported intraclass correlation coefficient was >0.90, “good” if 0.75 to 0.90, “moderate” if 0.50 to 0.74, and “poor” if <0.50. Between-limb differences of the operated and nonoperated limbs in the surgical group were calculated using a paired-samples *t* test or the Wilcoxon signed-rank test for normally and nonnormally distributed data, respectively. Between-group comparisons were performed using an independent *t* test and the Mann-Whitney *U* test for normally and nonnormally distributed data, respectively. Effect sizes were calculated using Cohen *d*. A subgroup analysis of surgical stabilization procedures was conducted using the same approach, although with effect sizes corrected using Hedges *g*. Pearson correlation coefficients (*r*) with 95% confidence intervals and the coefficient of determination (*R*^2^) were calculated to determine if any relationships existed between peak force and RFD measurements. Associations between measurements were interpreted as negligible (0.00-0.10), weak (0.11-0.39), moderate (0.40-0.69), strong (0.70-0.89), and very strong (0.90-1.00).^22^ All statistical analyses were performed using SPSS software (Version 29.0; IBM), with the level of significance set at *P*≤ .05.

## Results

A total of 60 patients (surgical group and control group) were referred for study participation and underwent testing between September 2021 and August 2024. Table 1 presents the characteristics for both groups. Both groups were well matched (Table 1).

**Table 1:** Participant Characteristics*^a^*

	Surgical Group (n = 30)	Control Group (n = 30)
Age, y	20.8 ± 3.3	21.3 ± 3.1
Height, cm	184.7 ± 6.1	185.4 ± 7.0
Weight, kg	81.2 ± 7.5	82.8 ± 8.1
Right limb dominance	28 (93.3)	27 (90.0)
Operated limb dominance	13 (43.3)	
Follow-up time, mo	4.0 ± 0.9	
Duration of symptoms, mo	7.0 ± 10.9	
No. of dislocations before surgery		
1	17 (56.7)	
2	8 (26.7)	
≥3	5 (16.6)	
Mechanism of injury		
Anterior contact	9 (30.0)	
Hyperflexion/abduction	8 (26.7)	
Horizontal hyperextension	10 (33.3)	
Force through elbow	3 (10.0)	
Type of surgery		
Arthroscopic anterior stabilization	6 (20.0)	
Arthroscopic anterior stabilization with remplissage	13 (43.3)	
Open Latarjet procedure	11 (36.7)	

a Data are shown as mean ± SD or n (%).

**Figure 2.**
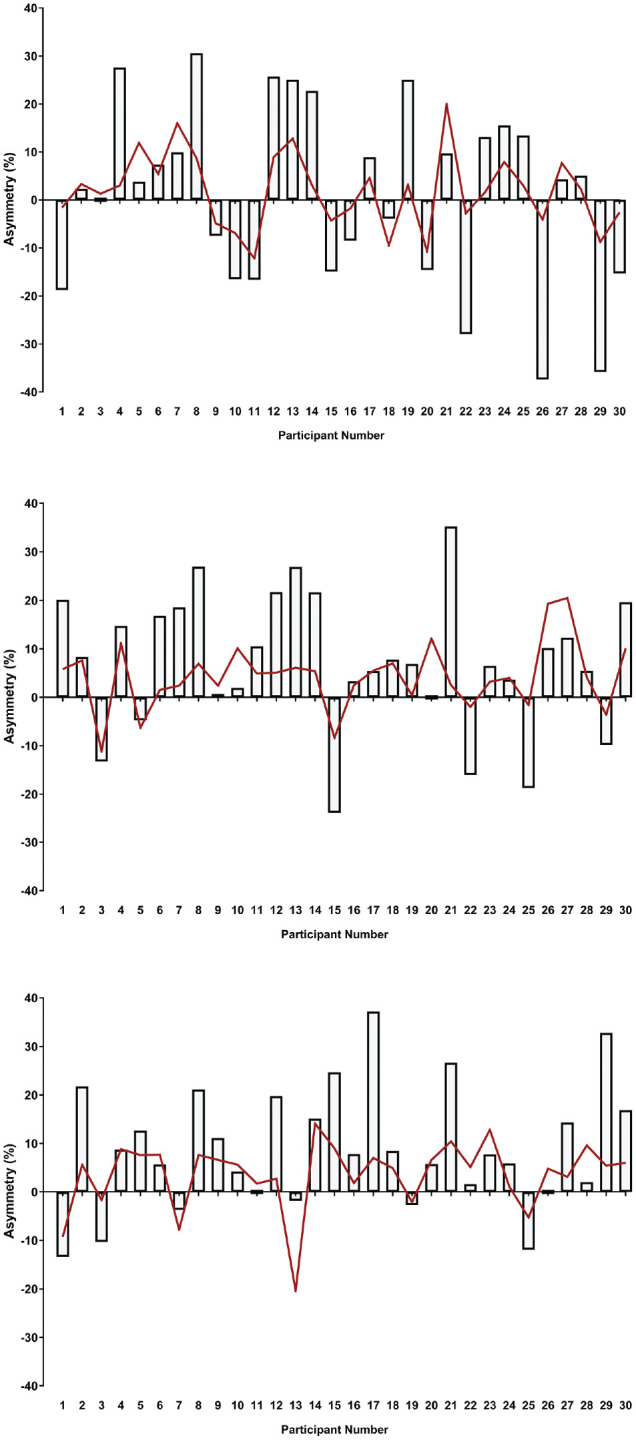
Peak force asymmetry data for all 30 Australian rules footballers after shoulder stabilization surgery in the (A) ISO-I, (B) ISO-Y, and (C) ISO-T test positions (>0 = asymmetry favors the nonsurgical arm; <0 = asymmetry favors the surgical arm). The individual coefficient of variation is shown by the red line.

Table 2 shows within-group comparisons of peak force between the surgical and nonsurgical arms of the stabilization group as well as between-group comparisons against the uninjured control group for all positions. In the ISO-I position, no statistically significant differences were observed between the surgical and nonsurgical arms of the stabilization group for peak force (Table 2). However, significant differences were observed between the surgical and control arms for peak force (*P* = .004; *d* = 0.74). In the ISO-Y position, peak force in the surgical arm was significantly less than peak force in the nonsurgical arm (*P* = .018; *d* = 0.28) as well as in the arm of the control group (*P* < .001; *d* = 0.96) (Table 2). Similarly, peak force in the ISO-T position was significantly less in the surgical arm than in the nonsurgical arm (*P* = .002; *d* = 0.37) as well as in the arm of the control group (*P* < .001; *d* = 1.19) (Table 2). Mean interlimb asymmetries in peak force of 1.1% ± 18.8%, 7.3% ± 14.2%, and 8.9% ± 12.5% were observed for the ISO-I, ISO-Y, and ISO-T positions, respectively. Figure 2 shows the highly individual and variable nature of interlimb asymmetry in peak force across the 3 test positions.

**Table 2:** Peak Force (in N)^*a*^

	Surgical Arm	Nonsurgical Arm	Surgical vs Nonsurgical		Control	Surgical Arm vs Control	
	Mean ± SD	Mean ± SD	*P*	Effect Size	Mean ± SD	*P*	Effect Size
ISO-I	115.7 ± 37.6	116.0 ± 33.0	.779	0.01	142.3 ± 34.4	**.004**	0.74
ISO-Y	90.2 ± 25.8	97.0 ± 23.2	**.018**	0.28	116.1 ± 27.9	**<.001**	0.96
ISO-T	83.6 ± 23.3	92.5 ± 24.9	**.002**	0.37	110.8 ± 22.3	**<.001**	1.19

a Bolded values indicate statistical significance (*P* < 0.05).

Table 3 shows within-group comparisons of the RFD at 100 and 200 milliseconds between the surgical and nonsurgical arms of the stabilization group as well as between-group comparisons against the uninjured control group for all positions. No significant differences were observed in the RFD over the first 100 milliseconds between the surgical arm and nonsurgical arm for any test position, nor were there any differences observed when compared with the uninjured control group (Table 3). Significant interlimb differences were observed between the surgical and nonsurgical arms for the RFD over the first 200 milliseconds in all test positions (Table 3). Similarly, significant differences in the RFD over the first 200 milliseconds were observed, with large effect sizes, between the surgical arm and the uninjured control group (Table 3).

**Table 3:** Rate of Force Development (in N/s)^*a*^

	Surgical Arm	Nonsurgical Arm	Surgical vs Nonsurgical		Control	Surgical Arm vs Control	
	Mean ± SD	Mean ± SD	*P*	Effect Size	Mean ± SD	*P*	Effect Size
ISO-I							
100 ms	221.1 ± 75.7	239.7 ± 77.1	.070	0.24	268.8 ± 111.7	.064	0.50
200 ms	263.5 ± 104.8	306.1 ± 107.3	**.007**	0.40	373.0 ± 120.3	**<.001**	0.97
ISO-Y							
100 ms	197.6 ± 61.8	208.1 ± 56.1	.371	0.18	226.1 ± 74.8	.127	0.41
200 ms	224.7 ± 81.7	261.5 ± 81.6	**.002**	0.45	304.1 ± 93.7	**<.001**	0.90
ISO-T							
100 ms	205.5 ± 59.9	216.3 ± 61.0	.280	0.18	211.6 ± 74.1	.328	0.09
200 ms	230.4 ± 79.4	269.6 ± 88.7	**.027**	0.47	304.8 ± 104.8	**.002**	0.80

a Bolded values indicate statistical significance (*P* < 0.05).

Strong significant correlations were observed between peak force and RFD over the first 200 milliseconds for all test positions, while moderate significant correlations were observed between peak force and RFD over the first 100 milliseconds for all test positions (Table 4). The between-trial measurement error was <10% for all positions and limbs for peak force in both groups, while the between-trial measurement error was >10% for all positions and limbs for the RFD at 100 and 200 milliseconds (see Appendix Tables A1 and A2). The relative reliability in the uninjured control group was good to excellent for all positions in both arms, except for the dominant arm in the ISO-Y position and the nondominant arm in the ISO-T position, which were moderate (see Appendix Table A1). The subgroup analysis comparing peak force and RFD between surgical stabilization procedures is presented in Appendix Table A3.

**Table 4:** Pearson Correlation Coefficients and Coefficients of Determination*^*a*^*

	Peak Force and RFD at 100 ms			Peak Force and RFD at 200 ms		
	*r*	*R* ^2^	*P*	*r*	*R* ^2^	*P*
ISO-I	0.678	0.460	**<.001**	0.794	0.630	**<.001**
ISO-Y	0.588	0.346	**<.001**	0.724	0.525	**<.001**
ISO-T	0.524	0.275	**.003**	0.841	0.707	**<.001**

a RFD, rate of force development. Bolded values indicate statistical significance (*P* < 0.05).

## Discussion

To our knowledge, this is the first study in which isometric peak force and RFD in the long lever position have been evaluated in Australian rules footballers after shoulder stabilization surgery, with outcomes compared with the nonoperated limb and a noninjured control group. Previous studies have shown high reinjury rates after shoulder stabilization surgery, particularly in young contact and collision athletes.^12,18^ Therefore, it is essential for clinicians to ensure that deficits in strength and performance are addressed before RTS.

Significant deficits in peak force and RFD were observed in the operated arm of Australian rules footballers at 4 to 6 months after stabilization surgery, partially supporting the first hypothesis. No significant differences in peak force were observed between limbs for the ISO-I position, and while significant interlimb differences were observed in the ISO-Y and ISO-T positions, effect sizes were only small to moderate. In contrast, large and very large effect sizes were observed for peak force in all test positions when comparing the operated limb to the uninjured control group. This is important when making decisions on RTS in that benchmarking peak force and RFD values to matched uninjured controls, or normative values, is preferred over an interlimb comparison. Of all ASH test positions, the ISO-T position demonstrated the greatest magnitude of interlimb and between-group differences. This position assessed tolerance to withstand long lever stress and the ability to transfer force across the shoulder girdle in horizontal hyperextension, which replicates the “arm tackle” position in sports such as Australian rules football or rugby.^4,28^ Indeed, this injury mechanism was the most common within the surgical group (33.3%), followed by anterior contact (30.0%), which occurred during marking and/or spoiling or because of landing with an outstretched arm (26.7%). This position also showed large between-group (although not interlimb) differences in peak force. Rehabilitation interventions should target maximal force production in these positions before RTS.

The current study demonstrated no differences in the RFD from 0 to 100 milliseconds (ie, early-phase RFD) for all 3 ASH test positions compared with the nonsurgical arm or the dominant arm of the uninjured control group. Conversely, significant differences were observed in the RFD from 0 to 200 milliseconds (late-phase RFD) for all 3 positions. Early-phase RFD is believed to reflect neural drive and intrinsic muscle properties, such as motor unit recruitment and the motor neuron discharge rate, whereas muscle size and maximal activation capacity (ie, strength) predominately influence the RFD within the first 200 milliseconds.^1^ This is supported in the present study in which peak force for all test positions was more strongly associated with the RFD from 0 to 200 milliseconds, demonstrating coefficients of determination percentages of 52% to 71%. In contrast, moderate correlations were observed with the RFD from 0 to 100 milliseconds, with only 27% to 46% of the variation in the RFD being explained by peak force. This is consistent with other studies that have reported a stronger explained variance with increases from the time of onset, including the knee^17^ and the hamstring.^26^ The findings in the present study suggest that these neural and intrinsic muscle factors, critical to early-phase RFD, remain unaffected in the surgically stabilized shoulder at 4 to 6 months after surgery compared with the nonsurgical arm as well as the dominant arm of an uninjured control group. An early RFD is influenced by the intrinsic properties of muscle fibers.^23^ One possible explanation could be that these properties are consistent in both healthy athletes and those who have experienced glenohumeral injuries and/or have undergone subsequent shoulder stabilization surgery. However, research on this in the upper limb is lacking, making the effects of shoulder stabilization surgery on neural and muscle contractile factors influencing the RFD speculative. Another possible explanation could be the general lack of athletic exercise programming that biases rapid force production with the upper limb in a long lever position. It is well established that muscle force improvements, in particular the RFD, are more significant when training exercises closely resemble the movement patterns used in testing and when performed with intent.^7,20^ While this explanation is also somewhat speculative, current consensus statements,^29^ while acknowledging the need for rehabilitation that addresses strength and power, do not highlight the importance of this being performed with the arm in long lever positions. Future research could include investigating whether resistance exercises prescribed at fast speeds, and performed with intent, can improve early-phase RFD.

While intertrial variability for peak force across all ASH test positions demonstrated good absolute and relative reliability, the RFD within the first 100 and 200 milliseconds showed absolute reliability of >10% in CV values in both the surgical and nonsurgical arms as well as the dominant and nondominant arms of the stabilization group and uninjured control group. The within-session reliability observed in this study is comparable with that in previous literature using the unilateral isometric midthigh pull and 90°-90° isometric hamstring assessments.^27^ While the RFD may provide good clinical information on the player’s capacity for explosive performance and joint protection,^4^ practitioners may need to be cautious of using this metric, given the high within-session variability.

Despite the good absolute reliability of peak force measurements across the 3 ASH test positions, interlimb asymmetries were highly individual and variable and did not always favor the nonsurgical arm, which is what was expected. When asymmetry values are considered, a threshold of between 10% and 15% is used to identify abnormal differences between limbs.^24^ Indeed, previous studies have shown that 84% of patients do not meet strength benchmarks at 6 months after surgery, as indicated by not achieving at least 90% of the strength of the nonoperated limb.^31^ Therefore, the mean limb asymmetry values in the current study can be considered small. However, as shown, large individual variability in limb asymmetry was present, which is most likely explained by arm dominance. Previous studies on upper limb asymmetries in patients with painful shoulders have shown that arm dominance can affect asymmetry values, with the limb symmetry index being approximately 17% lower in patients with nondominant shoulder involvement compared with those with dominant shoulder involvement.^25^ In the current study, no differences were observed in interlimb asymmetry of peak force between those with surgical stabilization on the dominant side compared with those with surgical stabilization on the nondominant side. However, this was most likely the result of the low sample sizes within these subgroups.

This is the first study to examine upper limb peak force and RFD in long lever positions in athletes after shoulder stabilization surgery. However, this study is not without limitations. First, limb dominance was not specifically accounted for when comparing the surgical limb with the control group. Instead, for between-group comparisons, the surgical arm was compared against the dominant arm of the control group. This approach, used similarly in anterior cruciate ligament literature,^19^ was intended to provide a more conservative estimate of the between-group differences especially because approximately 43% of the surgical group had their dominant limb stabilized. However, we acknowledge that this may influence the results, and future studies should consider limb dominance for more accurate comparisons. Second, while a key strength of the current study was the focus on male Australian rules footballers, who are a high-risk population for traumatic shoulder dislocations and reinjuries,^8^ it is uncertain whether these findings can be extended to female athletes, other collision and contact sports, or overhead sports that involve throwing. While the specific demands of Australian rules football are unique, the mechanisms of injury, such as high-velocity contact, multidirectional tackling, and overhead motion, are comparable with those seen in other sports such as rugby, American football, and Gaelic football. Future research that considers female athletes and other sporting contexts is required. Third, 3 different anterior shoulder stabilization procedures were included in this study, which adds heterogeneity to the surgical group and may have affected the results, although this heterogeneity is consistent with the common range of surgical management options for athletes with shoulder instability. While a subgroup analysis that compared peak force and RFD between arthroscopic and open Latarjet procedures was performed, the sample size was too small to find any meaningful and significant differences. Fourth, the physical therapy and rehabilitation regimen in the surgical group was not standardized, nor was it monitored for compliance or composition. We acknowledge that this may confound the surgical group’s results for peak force and RFD. However, it was also the intention of this preliminary study to recruit a consecutive series of community-level athletes undergoing rehabilitation with their own outpatient physical therapists, as would be routinely encountered in a clinical setting. Finally, the short follow-up time of 4 to 6 months may be considered a limitation, and longer term outcomes, including successful/nonsuccessful RTS and reinjuries, are indeed important. However, the aim of the current study was to characterize the current long lever isometric peak force and rapid force production profiles of a cohort of athletes at a time when they would ordinarily be considering RTS.

## Conclusion

This preliminary study of Australian rules footballers at 4 to 6 months after surgery exhibited significant deficits in long lever isometric peak force and RFD (0-200 milliseconds) compared with both their nonsurgical arm and the dominant arm of a matched, uninjured control group. Clinicians should recognize that these postoperative deficits exist but also consider the variability in values when using RFD as a performance metric as well as the limitations of interpreting limb asymmetry when assessing the extent of these deficiencies. Future studies should report on long lever isometric strength and RFD at longer follow-up time points and explore the associations between these measurements and patient-reported outcomes, RTS and performance, and rates of reinjuries as well as assess the influence of different stabilization procedures and postoperative rehabilitation.

## Appendix

**Table A1:** Within-Session Absolute and Relative Reliability for Control Group (n = 30)*^*a*^*

	Dominant Arm		Nondominant Arm	
	CV, %	ICC (95% CI)	CV, %	ICC (95% CI)
ISO-I				
Peak force	8.6	0.923 (0.858-0.961)	7.4	0.925 (0.862-0.962)
RFD 100 ms	16.4	0.861 (0.747-0.929)	17.1	0.834 (0.698-0.915)
RFD 200 ms	12.9	0.899 (0.816-0.949)	12.8	0.905 (0.825-0.952)
ISO-Y				
Peak force	5.3	0.969 (0.943-0.984)	6.7	0.916 (0.846-0.957)
RFD 100 ms	22.0	0.721 (0.486-0.859)	18.7	0.813 (0.658-0.905)
RFD 200 ms	13.2	0.867 (0.758-0.932)	10.5	0.938 (0.886-0.968)
ISO-T				
Peak force	6.3	0.940 (0.890-0.969)	6.1	0.940 (0.891-0.970)
RFD 100 ms	14.4	0.897 (0.813-0.948)	18.0	0.723 (0.498-0.858)
RFD 200 ms	10.9	0.937 (0.882-0.969)	11.9	0.933 (0.878-0.966)

a CV, coefficient of variation; ICC, intraclass correlation coefficient; RFD, rate of force development.

**Table A2:** Within-Session Absolute and Relative Reliability for Surgical Group at 4 Months (n = 30)*^*a*^*

	Surgical Arm		Nonsurgical Arm	
	CV, %	ICC (95% CI)	CV, %	ICC (95% CI)
ISO-I				
Peak force	6.5	0.972 (0.950-0.986)	6.9	0.955 (0.918-0.977)
RFD 100 ms	17.5	0.857 (0.740-0.927)	16.9	0.812 (0.654-0.905)
RFD 200 ms	13.0	0.960 (0.927-0.979)	13.6	0.905 (0.841-0.956)
ISO-Y				
Peak force	6.5	0.973 (0.950-0.986)	6.3	0.963 (0.933-0.981)
RFD 100 ms	16.7	0.806 (0.644-0.901)	17.4	0.732 (0.504-0.864)
RFD 200 ms	14.5	0.921 (0.854-0.960)	14.4	0.887 (0.793-0.942)
ISO-T				
Peak force	6.8	0.963 (0.931-0.981)	6.0	0.964 (0.933-0.982)
RFD 100 ms	17.4	0.765 (0.572-0.880)	16.2	0.825 (0.680-0.911)
RFD 200 ms	12.0	0.915 (0.844-0.957)	11.8	0.931 (0.874-0.965)

a CV, coefficient of variation; ICC, intraclass correlation coefficient; RFD, rate of force development.

**Table A3:** Peak Force and RFD for Arthroscopic Stabilization Versus Latarjet Procedures*^*a*^*

	Arthroscopic Stabilization (n = 19)		Latarjet (n = 11)		Arthroscopic Stabilization vs Latarjet	
	Involved Arm	Uninvolved Arm	Involved Arm	Uninvolved Arm		
	Mean ± SD	Mean ± SD	Mean ± SD	Mean ± SD	*P*	Effect Size
ISO-I						
Peak force, N	111.8 ± 34.1	114.2 ± 32.7	122.4 ± 43.9	119.2 ± 34.7	.470	−0.27
RFD 100 ms, N/s	202.9 ± 76.5	233.5 ± 78.1	252.5 ± 66.0	250.4 ± 78.1	**.042**	−0.66
RFD 200 ms, N/s	273.1 ± 115.0	317.6 ± 118.1	246.9 ± 87.0	286.3 ± 87.2	.260	0.28
ISO-Y						
Peak force, N	90.2 ± 25.8	97.0 ± 23.2	87.5 ± 20.9	92.5 ± 18.9	.330	0.16
RFD 100 ms, N/s	197.2 ± 69.8	212.1 ± 58.2	198.3 ± 47.8	201.1 ± 54.5	.482	−0.02
RFD 200 ms, N/s	240.4 ± 92.9	285.9 ± 84.3	197.5 ± 52.8	219.3 ± 58.8	.087	0.52
ISO-T						
Peak force, N	83.6 ± 23.3	92.5 ± 24.9	81.3 ± 19.8	94.2 ± 25.0	.344	0.15
RFD 100 ms, N/s	208.5 ± 62.6	214.8 ± 57.1	200.3 ± 57.4	218.8 ± 70.1	.362	0.13
RFD 200 ms, N/s	249.5 ± 82.6	280.1 ± 97.7	197.3 ± 63.8	251.5 ± 71.3	**.041**	0.67

a RFD, rate of force development. Bolded values indicate statistical significance (*P* < 0.05).
